# A Multi-Institutional Comparison of Dynamic Contrast-Enhanced Magnetic Resonance Imaging Parameter Calculations

**DOI:** 10.1038/s41598-017-11554-w

**Published:** 2017-09-11

**Authors:** Joint Head and Neck Radiotherapy-MRI Development Cooperative, Rachel B. Ger, Abdallah S. R. Mohamed, Musaddiq J. Awan, Yao Ding, Kimberly Li, Xenia J. Fave, Andrew L. Beers, Brandon Driscoll, Hesham Elhalawani, David A. Hormuth, Petra J. van Houdt, Renjie He, Shouhao Zhou, Kelsey B. Mathieu, Heng Li, Catherine Coolens, Caroline Chung, James A. Bankson, Wei Huang, Jihong Wang, Vlad C. Sandulache, Stephen Y. Lai, Rebecca M. Howell, R. Jason Stafford, Thomas E. Yankeelov, Uulke A. van der Heide, Steven J. Frank, Daniel P. Barboriak, John D. Hazle, Laurence E. Court, Jayashree Kalpathy-Cramer, Clifton D. Fuller

**Affiliations:** 1 Department of Radiation Physics, The University of Texas MD Anderson Cancer Center, Houston, Texas USA; 2 The University of Texas MD Anderson Cancer Center UTHealth Graduate School of Biomedical Sciences, Houston, Texas USA; 3 Department of Radiation Oncology, The University of Texas MD Anderson Cancer Center, Houston, Texas USA; 4 Department of Clinical Oncology and Nuclear Medicine, Faculty of Medicine, University of Alexandria, Alexandria, Egypt; 5 Case Western Reserve University, Cleveland, OH USA; 6 University Hospitals, Cleveland, OH USA; 7 Department of Imaging Physics, The University of Texas MD Anderson Cancer Center, Houston, Texas USA; 8 Advanced Imaging Research Center, Knight Cancer Institute, Oregon Health & Science University, Portland, Oregon USA; 9 The International School of Beaverton, Beaverton, Oregon USA; 10 Athinoula A. Martinos Center for Biomedical Imaging, Massachusetts General Hospital/Division of Health Sciences & Technology, Massachusetts Institute of Technology, Charlestown, Massachusetts USA; 11 Techna Institute, University Health Network, Toronto, Ontario Canada; 12 Institute for Computational Engineering and Sciences, The University of Texas, Austin, Texas USA; 13 Department of Radiation Oncology, The Netherlands Cancer Institute, Amsterdam, The Netherlands; 14 United Imaging Healthcare America, Houston, Texas USA; 15 Department of Biostatistics, The University of Texas MD Anderson Cancer Center, Houston, Texas USA; 16 Radiation Medicine Program, Princess Margaret Cancer Centre, University Health Network, Toronto, Ontario Canada; 17 Department of Radiation Oncology, University of Toronto, Toronto, Ontario Canada; 18 Department of Otolaryngology Head and Neck Surgery, Baylor College of Medicine, Houston, Texas USA; 19 Department of Head and Neck Surgery, The University of Texas MD Anderson Cancer Center, Houston, Texas USA; 20 Department of Molecular and Cellular Oncology, The University of Texas MD Anderson Cancer Center, Houston, Texas USA; 21 Department of Radiology, Duke University Medical Center, Durham, North Carolina USA

## Abstract

Dynamic contrast-enhanced magnetic resonance imaging (DCE-MRI) provides quantitative metrics (e.g. K^trans^, v_e_) via pharmacokinetic models. We tested inter-algorithm variability in these quantitative metrics with 11 published DCE-MRI algorithms, all implementing Tofts-Kermode or extended Tofts pharmacokinetic models. Digital reference objects (DROs) with known K^trans^ and v_e_ values were used to assess performance at varying noise levels. Additionally, DCE-MRI data from 15 head and neck squamous cell carcinoma patients over 3 time-points during chemoradiotherapy were used to ascertain K^trans^ and v_e_ kinetic trends across algorithms. Algorithms performed well (less than 3% average error) when no noise was present in the DRO. With noise, 87% of K^trans^ and 84% of v_e_ algorithm-DRO combinations were generally in the correct order. Low Krippendorff’s alpha values showed that algorithms could not consistently classify patients as above or below the median for a given algorithm at each time point or for differences in values between time points. A majority of the algorithms produced a significant Spearman correlation in v_e_ of the primary gross tumor volume with time. Algorithmic differences in K^trans^ and v_e_ values over time indicate limitations in combining/comparing data from distinct DCE-MRI model implementations. Careful cross-algorithm quality-assurance must be utilized as DCE-MRI results may not be interpretable using differing software.

## Introduction

Head and neck squamous cell carcinoma (HNSCC) is the sixth most common cancer worldwide^1^. Its 5-year survival rate has failed to improve from about 60% despite advances in imaging, surgery, radiotherapy targeting, and chemotherapy^2^. Thus, researchers are striving to individualize therapy for HNSCC to improve survival rates while limiting toxic effects in normal tissue, such as xerostomia, which can impact a patient’s quality of life. Dynamic contrast-enhanced magnetic resonance imaging (DCE-MRI) is a noninvasive tool for examination of the microvasculature of tumors and normal tissue. The perfusion and permeability metrics estimated from pharmacokinetic modeling of DCE-MRI data may provide an indirect measure of tumor hypoxia, a condition associated with poor prognosis in HNSCC^3,4^. Therefore, it may be possible to build prognostic models to help tailor HNSCC treatments to individual patients based on that patient’s DCE-MRI signature.

Investigators have used DCE-MRI to assess therapeutic response of HNSCC and have shown associations between DCE-MRI metrics and changes in salivary glands and mandible^5–11^. To the best of our knowledge, its use as a prognostic tool to inform treatment decisions for HNSCC has yet to be investigated in a large multisite prospective trial. Before such trials can begin, DCE-MRI inter-algorithm comparisons must be conducted to ensure consistency of output parameter maps for collating data during the multi-institution trial. Two quantitative metrics for DCE-MRI are the transfer constant for contrast agent transport from the blood plasma into the extravascular extracellular space (K^trans^) and the volume fraction of the extravascular extracellular space (v_e_). The calculation of these quantitative metrics can be impacted by the acquisition parameters. The accuracy and precision of these quantitative metrics can be influenced by arterial input function (AIF) quantification, temporal resolution in data acquisition, signal-to-noise ratio (SNR), and pharmacokinetic model selection^12–22^. For example, uncertainties in T1 map values and applied flip angle have been reported to cause errors of 88% in K^trans^ and 73% in v_e_, while reduced temporal resolution by 7-fold have reported decreses in K^trans^ up to 48%^19^. Therefore, acquisition parameters must be thoroughly tested and uniform across patients as they can dramatically impact measured DCE-MRI parameters.

The Tofts-Kermode pharmacokinetic model^23^ is the most commonly used model for DCE-MRI analysis, but implementation of each algorithm differs in facets such as data preprocessing, approaches to numerical optimizations in kinetic analysis, and data postprocessing, which may impact the values of the output quantitative metrics. Several recent studies demonstrated significant inter-algorithm variability when evaluating DCE-MRI of the female pelvis, breast, and rectum^24–26^. Of these studies, the one by Huang *et al*.^25^ demonstrated systematic differences in output parameter values between algorithms, which meant that results from different algorithms could be used together if correction factors were applied; the other studies, however, did not demonstrate any systematic errors. In addition, Cron *et al*.^27^ found that the percentage of nonphysical values (e.g. v_e_ values greater than 1) in the quantitative metrics increased as noise increased when they tested using three software packages. This noise dependence and inter-algorithm variance in quantitative DCE-MRI metrics are large obstacles to the clinical implementation of DCE-MRI and must be thoroughly investigated before proceeding with large multisite clinical trials using DCE-MRI in HNSCC patients.

In this study, we investigated the variability in K^trans^ and v_e_ across algorithms that are based on the Tofts-Kermode and extended Tofts pharmacokinetic models^28,29^. For this purpose, we used digital reference objects (DROs) from the Radiological Society of North America Quantitative Imaging Biomarkers Alliance^30^ and DCE-MRI data from oropharyngeal squamous cell carcinoma patients who underwent multiple DCE-MRI scans during treatment with definitive chemoradiotherapy.

## Results

### DROs

**Figure 1:**
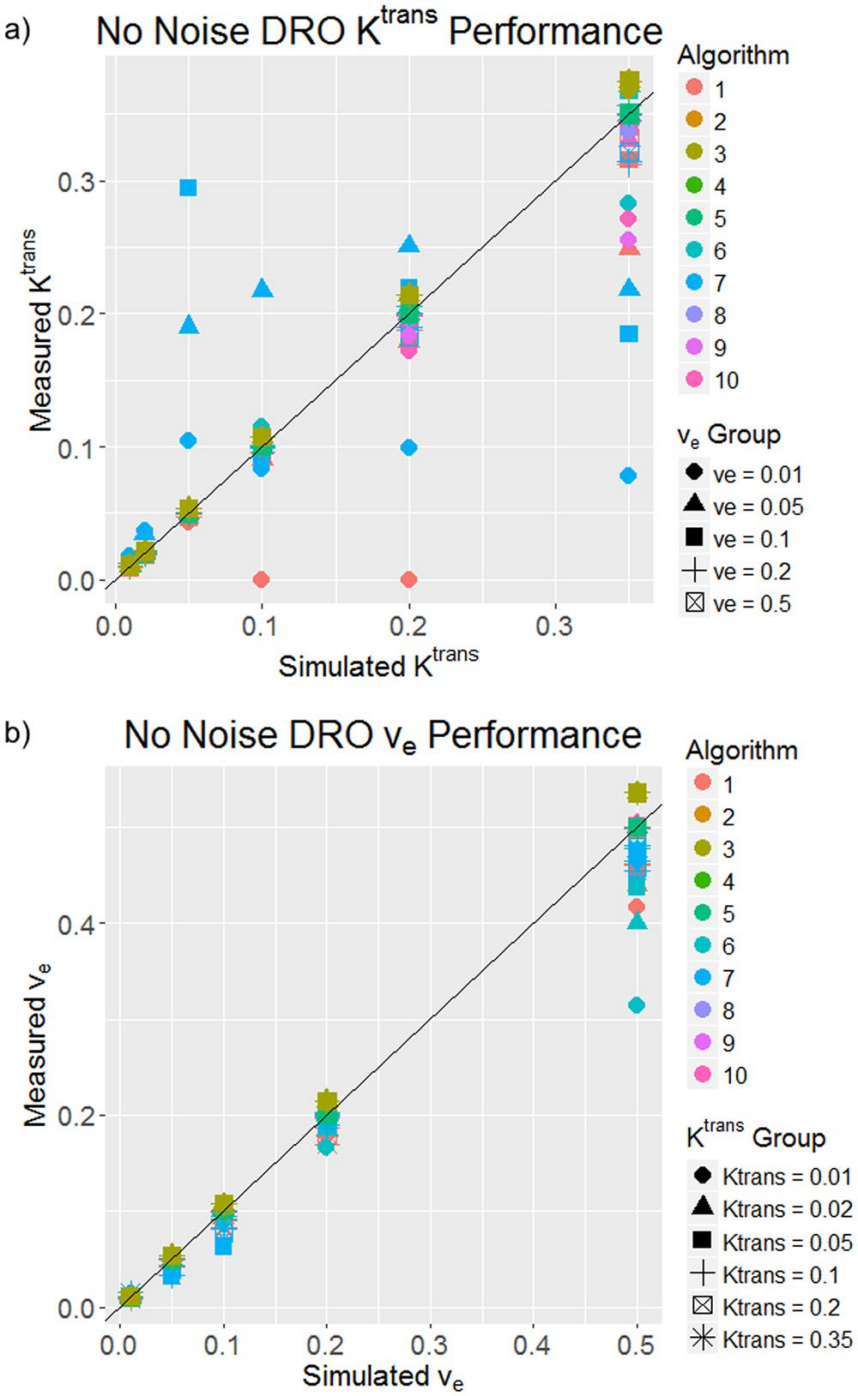
Plots of algorithm performance in a DRO with no noise for (**a**) K^trans^ and (**b**) v_e_. The simulated values are on the x-axis, and the measured values from each algorithm are on the y-axis. The 45° line represents 100% accuracy of the measured values. Each color represents a different algorithm, and each shape represents a different v_e_ column in (**a**) and a different K^trans^ row in (**b**).

One of the Tofts-Kermode algorithms (algorithm 11) could not process the DROs because of the algorithm’s structure. Therefore, the remaining 10 algorithms were used for DRO analysis. For the noiseless DRO, the stratified permutation test demonstrated that both K^trans^ and v_e_ were statistically significantly ordered correctly (p < 0.05) for all of the algorithms. Eighty-two percent of pairwise algorithm comparisons were statistically significantly different (p < 0.05) regarding K^trans^, and 69% of the comparisons were statistically significantly different (p < 0.05) regarding v_e_ based on the Wilcoxon rank-sum test. Figure 1 shows the algorithm performance for the noiseless DRO. Most of the K^trans^ and v_e_ measured values in the noiseless DRO were close to the true simulated values: 96% of K^trans^ and 96% of v_e_ measured values were within 10% of the simulated values. More spread in the measured values was observed at higher simulated values of K^trans^ or v_e_. Heat maps of the percentage error of K^trans^ and v_e_ measured values in comparison to the simulated values are shown in the supplemental material (Supplemental Fig. 1).

The stratified permutation test for the 28 DROs with noise demonstrated that in 86% and 84% of the cases (algorithm-DRO combinations), K^trans^ and v_e_ were statistically ordered correctly (p < 0.05) when one of the algorithms was excluded because of missing K^trans^ values and failure of the v_e_ test for all 28 of these DROs. Most of the test failures occurred at the lowest SNR (0.18). Eighty-four percent of the K^trans^ pairwise comparisons and 81% of the v_e_ pairwise comparisons were statistically significantly different (p < 0.05) based on the Wilcoxon rank-sum test results.

**Figure 2:**
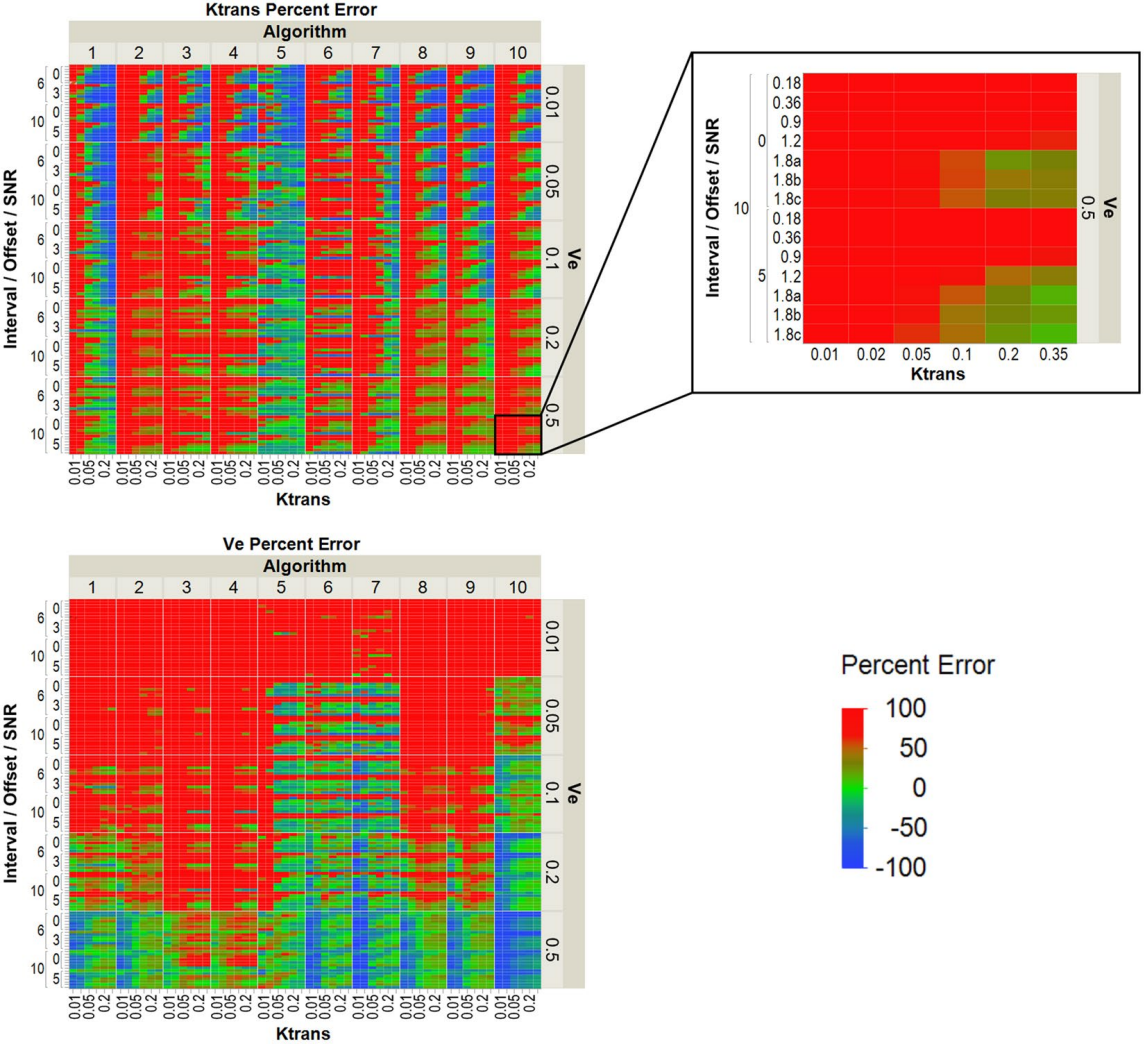
Heat maps of the percentage error for K^trans^ (top left) and v_e_ (bottom left) by algorithm in the 28 DROs with noise. The percentage error is defined using the formula ([measured − simulated]/simulated *100). The left side of the heat map is grouped by the timing interval used for the DRO (6 or 10 s), the timing offset used for the DRO (0 or 3 s for the 6 s timing interval, 0 or 5 s for the 10 s timing interval), and the SNR (0.18–1.8). The inset (top right) shows the K^trans^ and SNR values for each block in the heat maps. The maximum percentage error is defined as 100%, and the minimum percentage error is set to −100%. Any errors greater than the maximum percentage are also mapped as 100% error in color. Each DRO is differentiated by its sampling interval, timing offset, and SNR as determined by the S_0_ and sigma value used to create the DRO.

Heat maps of the percent error in K^trans^ and v_e_ relative to the simulated values in the 28 DROs with noise are shown in Fig. 2. The maximum percent error in this figure was set to 100% and the minimum percent error was set to −100%. Therefore, any K^trans^ and v_e_ values greater than the maximum percent error are mapped to red. The only trend found was less error at higher K^trans^ and v_e_ simulated values although there is more spread in the measured values at these higher K^trans^ and v_e_ simulated values. Algorithms that used spatial averaging were found to have statistically significantly less (p < 0.05) K^trans^ and v_e_ calculated error than algorithms that did not have spatial averaging according to the student’s t-tests.

We observed large variation in the percentage of values removed due to the threshold for K^trans^ and v_e_ for each algorithm. Some algorithms had almost no values removed, and some had a median of 70% of values removed.

These DRO results are for one method of excluding K^trans^ and v_e_ values. We also analyzed the data using the central 95% of the data for each K^trans^-v_e_ pair with no threshold restrictions, which produced consistent test results.

### Patients

**Figure 3:**
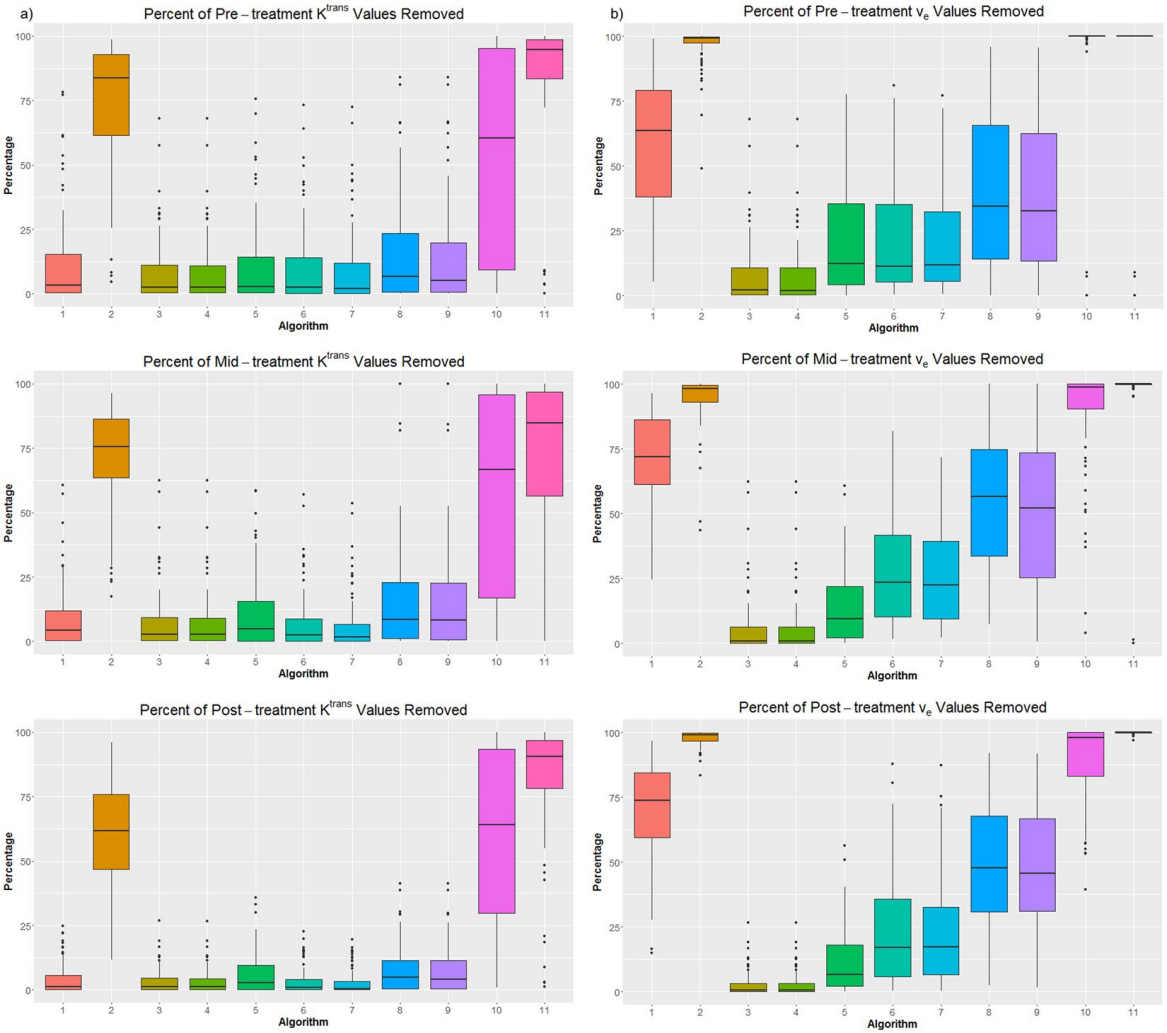
Percentages of (**a**) K^trans^ and (**b**) v_e_ values removed from patient images. The boxplots for each algorithm include the percentages removed for all patients and contours.

The percentages of K^trans^ and v_e_ values removed from patient ROIs because they were outside the bounds of the threshold are shown in Fig. 3 for the pretreatment, midtreatment, and posttreatment K^trans^ and v_e_. As in the DROs, the percentages varied: some algorithms had low percentages removed, implying that they mostly produced realistic values, whereas some algorithms produced almost nothing but unrealistic values for certain patients. The average percentage removed for K^trans^ was 27%, 26%, and 22% for pretreatment, midtreatment, and posttreatment respectively. The average percentage removed for v_e_ was 46%, 49%, and 48% for pretreatment, midtreatment, and posttreatment respectively.

According to results of the likelihood ratio test, all differences were statistically significantly (p < 0.05) dependent upon the algorithm except for the pretreatment-to-posttreatment change in K^trans^ when all algorithms were included in the model. Algorithms were subset into Tofts-Kermode and extended Tofts groups. In the Tofts-Kermode group, three changes were not statistically significantly dependent on algorithm (p < 0.05): pretreatment-to-midtreatment change in K^trans^, midtreatment-to-posttreatment change in K^trans^, and midtreatment-to-posttreatment change in v_e_. In the extended Tofts group, algorithm was not a significant factor (p < 0.05) in pretreatment-to-posttreatment change. In all other changes, the algorithm was a significant factor. In all linear mixed effects models, the variance explained by the ROI was much smaller than the residual variance, suggesting that the ROI does not explain much of the variation seen in the linear mixed effects model. All organ variance was less than 30% of the residual variance; on average, it was 8% of the residual variance.

**Figure 4:**
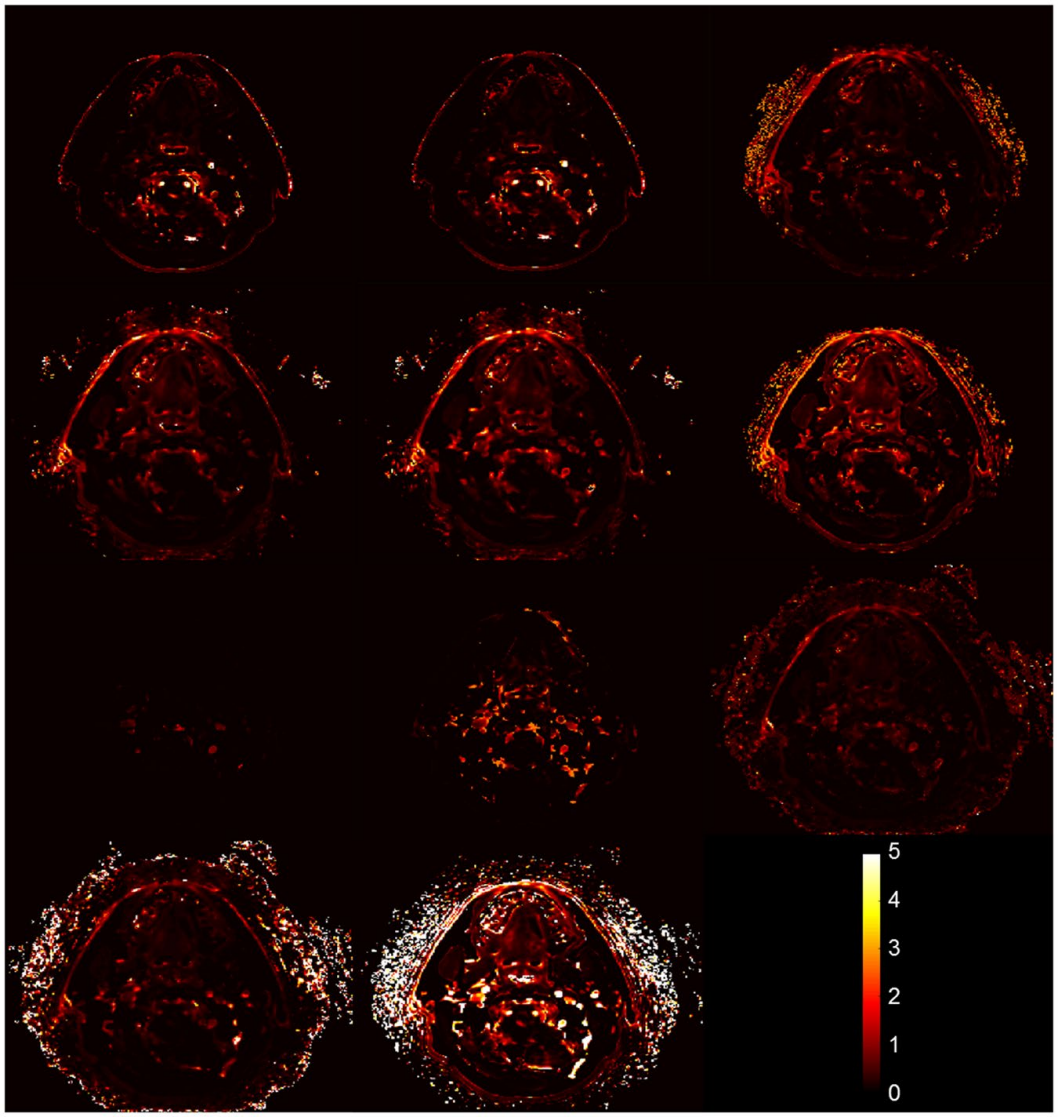
Illustration of differences in K^trans^ (min^−1^) maps exported by different algorithms for one axial DCE-MRI slice.

Figure 4 demonstrates an example of the difference in parameter values exported from different algorithms. The K^trans^ maps from the same axial slice of a patient are shown for all algorithms. It can be seen that some algorithms output mostly lower K^trans^ values while others output mostly higher K^trans^ values. In addition, some algorithms fit the noise data in voxels outside of the anatomy while other algorithms generated K^trans^ maps only within the anatomy.

Carletta’s thresholds for good agreement between algorithms (α ≥ 0.8) and sufficient agreement for tentative conclusions (0.800 > α > 0.667) were used^31^ to assess the results of Krippendorff’s alpha tests. The tests were run using all of the algorithms and also subsets of the algorithms, which were placed into Tofts-Kermode and extended Tofts groups. Of all of these tests, only those in the extended Tofts group had alphas that fell in range for tentative conclusions: 7 of the 108 tested correlations in this group had alphas in this tentative conclusions range. No alphas were in the good agreement range. An illustration of this inconsistent sorting of patients is shown in the supplemental material (Supplemental Fig. 3). Carletta’s thresholds for good agreement and tentative conclusions are weaker than those suggested by others. Krippendorff^32^ and Neuendorf^33^ suggested using higher standards, which would remove all the metrics found to be partially reliable across algorithms.

Few statistically significant Spearman correlations (p < 0.05) were observed: 8% of all tested K^trans^ correlations and 29% of all tested v_e_ correlations across all aglorithms. The only trend in these correlations across algorithms was a statistically significant Spearman correlation of v_e_ in the GTV-P.

## Discussion

Use of DCE-MRI is increasing in oncology research and investigators have performed many promising studies indicating correlations between predicted therapeutic outcome and DCE-MRI metrics^7,8^. However, many different DCE-MRI platforms were employed in these studies, and no studies have demonstrated whether data and conclusions regarding HNSCC can be aggregated. We addressed this issue by analyzing the same sets of DRO and HNSCC patient data with a subset of the currently used algorithms that are based on the Tofts-Kermode or extended Tofts model, as these pharmacokinetic models are the ones most commonly employed in DCE-MRI.

The key results from this study are that algorithms were able to determine high values from low values on DROs, but workflow differences may obscure the ability to discern values across algorithms in patients. This may be specifically related to T1 mapping which was not controlled in the patient portion of this study. Specifically, trends among algorithms from the same institution (institution supplied both Tofts-Kermode and extended Tofts algorithms) were consistent, but not across institutions. This highlights the effect of preprocessing, also shown by the impact of spatial averaging on the calculated error. Therefore, translatability of DCE-MRI across algorithms is not currently feasible.

A digital phantom was used to assess algorithms with a known “ground truth”. The DROs we used had SNRs of 0.18 to 1.80 in the noisy DROs. Although these SNR values and K^trans^ and v_e_ values within the DRO are below that typically found in head and neck cancer cases^34–37^, the DROs were used due to their availability and Quantitative Imaging Biomarkers Alliance-backed quality. The DROs, however, do not come with instructions for interpretation of results, which makes conclusions difficult especially for the DROs that contain very high noise.

The good algorithm performance for the noiseless DRO is consistent with the results reported by Huang *et al*.^25^ and suggest that the algorithms tested here are constructed properly. However, the error increased dramatically when high levels of noise were added to the images. Our assessment using percentage error may explain why the error appeared extremely high in the low K^trans^ and v_e_ regions as a small absolute error in this region will appear with a high percentage error. Heat maps of the error with the noisy DROs are shown in the supplementary data (Supplemental Fig. 2) to remove this discrepancy in percentage error between low and high values.

The difference between algorithms was significant for DROs according to the Wilcoxon rank-sum test results, which is consistent with the results reported by Beuzit *et al*.^26^, who used SNRs of 10 and 100 and still found significant differences between different software packages. A limitation of this test is that if the differences between two algorithms are small but all of one sign (such that all values from one algorithm are higher than all values from another algorithm), the differences will be statistically significant. This does not appear to be the cause of the statistically significant differences observed here because each algorithm has its own error signature, and we could not identify a systematic error in any of the algorithms.

The DRO results demonstrated the potential of DCE-MRI quantitative metrics for clinical application, an illustration of which was the patient data set we used. The significance of including algorithm in the linear mixed effects models was consistent with the Wilcoxon rank-sum test results for the DROs. The small variance explained by the ROI compared with the residual variance in the linear mixed effects models was surprising. If associations can be found using DCE-MRI, different trends between normal tissue and tumor would particularly be expected, yet the ROI provided little explanation of the variance in the data in the linear mixed effects models.

A majority of the algorithms tested produced statistically significant Spearman correlations of v_e_ in the GTV-P. The agreement of Spearman correlations across algorithms within the GTV-P but not within normal tissue may be due to a difference in contrast-induced signal change, as the GTV-P has a much higher signal change than does normal tissue in DCE-MRI. This means that the GTV-P has higher K^trans^ and lower error in the presence of noise based on the DRO data. However, this agreement of Spearman correlations of v_e_ in the GTV-P is contradicted by the Krippendorff’s alpha results for the GTV-P. Only the midtreatment K^trans^ value in the extended Tofts group had an alpha in the range where tentative conclusions can be drawn. This discrepancy may be explained by small interpatient variability in the K^trans^ and v_e_ values, which limited the algorithms’ ability to separate patients into above or below the median. However, the Spearman rank correlation coefficient identifies trends and is not as affected by interpatient differences in values as Krippendorff’s alpha if the trend is consistent.

The Krippendorff’s alpha results demonstrated that different algorithms do not consistently classify patients’ K^trans^ and v_e_ values, change in values, or percent change in values. These results indicate that there is currently no clinical level at which these quantitative metrics can be used across algorithms to quantify patients. Based on the algorithms’ performance for the DROs in the stratified permutation test in our study, this result from Krippendorff’s alpha tests is surprising. However, small interpatient variation in the K^trans^ and v_e_ values may have caused the low inter-algorithm reliability. This low inter-algorithm reliability, even within the Tofts-Kermode and extended Tofts groups, contrasts with the results described by Huang *et al*.^25^. They found good parameter agreement for percentage change when they grouped algorithms by pharmacokinetic model and that all of the algorithms provided good prediction of response to therapy as assessed using univariate logistic regression. This difference may have resulted from the imaging technique used, tissue of interest, and/or patient distribution of K^trans^ and v_e_ values.

Uncertainties in DCE-MRI exist due to AIF selection and imaging parameters^12,13,16–18,22^, but we did not explore them in this study because they were controlled: we examined each algorithm with the same patient DCE-MRI images, variable-flip-angle images, and AIF. In previous studies, T1 mapping and AIF selection impacted K^trans^ and v_e_ values^12–17,22,38–40^. The agreement between algorithms that we observed may have been lower if we had included all of the differences typically seen in a multisite clinical trial, including different scanners, scanning protocols, AIFs, DCE-MRI algorithms at each institution. In our relatively controlled study, we observed statistically significant differences in both DRO and patient data among the algorithms. It must be acknowledged that there is no ‘ground truth’ against which these algorithms can be compared, and it is unclear whether there was a true therapeutic effect that should have been identified by DCE-MRI of patient data. Even if there was no net effect across this population of patients, however, it is clear that different approaches to DCE-MRI analysis have significant impact on within-patient trends.

We chose the upper bound for K^trans^ since one of the algorithms in this study used 5 min^−1^, providing a feasible physical upper limit. We chose the lower bound for K^trans^ because when a given pixel or voxel has a poor fit within an algorithm, it is often given a value of 0 or a negative value. Accordingly, we excluded these values from analysis. We chose the bounds for v_e_ based on the physical limits given by its definition as a fractional space. Furthermore, poor fits in an algorithm are often mapped to 0 or 1. Therefore, we excluded these values. While 0 is a physically realistic value for K^trans^ and 0 and 1 are physically realistic values for v_e_, these values must be excluded owing to a high proportion of bad pharmacokinetic model fits mapped to these values. The high percentage of values that must be removed represents an area of improvement for future algorithms. Cron *et al*.^27^ demonstrated that as noise in DCE-MRI scans increases, the percentage of nonphysical K^trans^ and v_e_ values increases. Thus, voxel-based analysis of DCE-MRI quantitative metrics may not be reliable, so global metrics, such as average, of regions must be used for studies. For regions in which a high percentage of values are excluded, the average value extracted is not a reliable metric, as it comes from only a small subregion which is not representative of the whole region. This issue can be mitigated on the imaging end by increasing the SNR at the cost of the increased scan time, poorer temporal resolution, spatial resolution, or coverage, and potentially on the software end by improving how algorithms handle noise through the use of DROs.

In summary, we showed that rigorous standardization and careful quality assurance of software programs, including comparison of parameter calculations with standard data sets, are needed for collating pharmacokinetic analysis of DCE-MRI data among different algorithms. This must include assessment of the impact of image noise on quantitative metric error. Authors recently reported the need for careful quality assurance for functional MRI^41^. Efforts like those by the Quantitative Imaging Biomarkers Alliance to standardize DCE-MRI acquisition parameters represent a natural step forward for quality assurance and serve as the foundation for the current quality assurance work used in the present study.

To support these efforts, we provided our data set in a repository to allow for their use as perpetual head and neck cancer patient-derived standards for future DCE-MRI software and/or algorithm development in addition to the extant DRO library maintained by one of the authors (D. Barboriak). To that end, we recommend the following:

1. Consistent use of the same software for DCE-MRI analysis within a given study and for cross-comparisons between studies.
2. Specification and setting of acquisition parameters before proceeding with clinical trials as with the present data set.
3. Before performing multi-institution clinical trials, confirmation that DCE-MRI parameter values are consistent across institutions.
4. Inclusion of reference to a DRO with clinically relevant SNRs to benchmark performance of DCE-MRI software using clear evaluation criteria.

Clinically, our DRO data point to the fact that algorithms differed substantially despite reliance on the same basic underlying pharmacokinetic model(s), performing relatively stable in low-noise conditions. This, coupled with the inter-algorithm variability observed with the *in vivo* head and neck cases (which were performed in immobilization on a single MRI platform with standard AIF selection) suggests that, at present, any clinical trial desiring to implement DCE-MRI, should at a minimum, use a single pre-specified DCE-MRI software workflow, and eschew use of multiple algorithms. This also means that DCE-MRI findings from one software are broadly uninterpretable in a differing platform at present.

Until quantitative metrics can be reliably calculated across algorithms, patient-derived DCE-MRI analyses with different algorithms cannot be aggregated. Semiquantitative metrics, such as the area under the curve, have been shown to be more reproducible than quantitative metrics and may be the best interim option for use in prognostic studies using different algorithms^42^. Further refinement is required before DCE-MRI software-derived parameters can be used as a routine cross-institutional metric for multi-site clinical trials.

## Materials and Methods

### Algorithms

Eleven algorithms from six institutions and one commercial software package were analyzed. They consisted of seven Tofts-Kermode models (identified herein as algorithms 2, 3, 5, 6, 8, 10, 11) and four extended Tofts models (algorithms 1, 4, 7, 9). Spatial averaging on the DCE-MRI images was used in algorithms 5, 6, 7, 8, and 9. All algorithms are currently used for research applications at the respective institutions. The algorithms are described in Table 1.

**Table 1:** Description of Algorithms.

Institution	Model(s) Used
Massachusetts General Hospital	Tofts-Kermode (description in Supplemental Data)
MD Anderson Cancer Center	Tofts-Kermode and Extended Tofts (description in Supplemental Data)
Netherlands Cancer Institute	Tofts-Kermode and Extended Tofts^47^
nordicICE	Extended Tofts^48^
Oregon Health & Science University	Tofts-Kermode and Tofts-Kermode^14,49,50^
Princess Margaret Cancer Center	Tofts-Kermode and Extended Tofts^51,52^
University of Texas at Austin	Tofts-Kermode^53,54^

Algorithms are listed in alphabetical order not order displayed in figures.

### DROs

DROs provided by the Radiological Society of North America Quantitative Imaging Biomarkers Alliance were used to assess algorithm performance. The DROs had six K^trans^ values ranging from 0.01 min^−1^ to 0.35 min^−1^ that were constant across the rows and five v_e_ values ranging from 0.01 to 0.5 that were constant down the columns, resulting in 30 different K^trans^-v_e_ pairs, each encompassing 10 × 10 pixels. The K^trans^ and v_e_ values were used to generate synthetic image data using the Tofts-Kermode two-parameter model run in JSim, an open-source modeling system^23,43^. One DRO without noise^44^ and 28 DROs with noise (SNR 0.18–1.8)^45^ simulated by varying the sampling interval, timing offset, S_0_, and sigma value were used to evaluate algorithm performance. For each K^trans^-v_e_ pair, the output pixels from the algorithms were subjected to a threshold to non-physiologic pixels (0 < K^trans^ output <5 and 0 < v_e_ output <1) and then averaged.

### Patients

Fifteen patients diagnosed with human papillomavirus-positive oropharyngeal squamous cell carcinoma were included in this study under a protocol approved by the institutional review board at MD Anderson Cancer Center. All patients gave their study-specific informed consent. All methods were performed in accordance with the relevant guidelines and regulations. Patients underwent DCE-MRI scans from December 2013 to October 2014. The criteria for study inclusion were an age older than 18 years, histologically documented stage III or IV human papillomavirus-positive oropharyngeal squamous cell carcinoma according to the American Joint Committee on Cancer 7^th^ edition staging criteria, eligibility for definitive chemoradiotherapy, and an Eastern Cooperative Oncology Group performance status of 0 to 2. Patients were excluded for any of the following reasons: definitive resection of a primary tumor, administration of induction chemotherapy before radiotherapy, a prior cancer diagnosis except that of appropriately treated localized epithelial skin cancer or cervical cancer, prior radiotherapy to the head and neck, contraindications for gadolinium-based contrast agents, and claustrophobia.

Patient median age was 56 years (range, 47–68), with 14 men and 1 woman. All patients received radiotherapy at 70 Gy in 33 fractions. The majority of the patients (87%) received cisplatin-based chemotherapy concurrently with radiotherapy. Patient, disease, and treatment characteristics are listed in Table 2. Patient 12 did not have a primary tumor because he underwent bilateral tonsillectomy before scanning.

**Table 2:** Study Patient Demographics.

Patient Number	Sex	Age (years)	Race/ Ethnicity	Smoking Status	Primary Tumor Site	TNM Category	Chemotherapy (weekly)
1	M	52	White	N	Base of tongue	T3N1M0	Cisplatin
2	M	53	White	Y	Base of tongue	T2N2aM0	Cetuximab
3	M	60	White	Y	Tonsil	T4N2bM0	Cisplatin
4	M	55	White	Y	Tonsil	T3N2bM0	Cisplatin
5	M	65	White	N	Base of tongue	T2N1M0	Cetuximab
6	M	57	Hispanic	Y	Tonsil	T2N2cM0	Cisplatin
7	M	60	White	Y	Base of tongue	T2N2bM0	Cisplatin
8	M	58	Black	Y	Base of tongue	T2N2cM0	Cisplatin
9	M	62	Asian	Y	Tonsil	T4N2cM0	Cisplatin
10	F	48	White	Y	Tonsil	T4N2bM0	Cisplatin
11	M	56	White	N	Tonsil	T2N2cM0	Cisplatin
12	M	68	White	Y	Tonsil	TxN2cM0	Cisplatin
13	M	47	White	N	Tonsil	T3N2bM0	Cisplatin
14	M	47	White	Y	Tonsil	T3N2bM0	Cisplatin
15	M	55	White	N	Base of tongue	T4N2bM0	Cisplatin

All patients underwent DCE-MRI scans within 1 week prior to treatment, 3–4 weeks after the start of treatment, and 6–8 weeks after the completion of treatment. The DCE-MRI scans were done using a 3.0 T Discovery 750 MRI scanner (GE Healthcare) with six-element flex coils and a flat insert table (GE Healthcare). The same immobilization devices (individualized head and shoulder mask, customized head support, and intraoral tongue-immobilizing/swallow-suppressing dental stent) were employed in longitudinal scans to improve image co-registration and to reduce interval physiologic motion (e.g., swallowing).

Thirty axial slices with a field of view of 25.6 cm and thickness of 4 mm were selected to cover the spatial region encompassing the palatine process region cranially to the cricoid cartilage caudally for all scans. Prior to DCE-MRI, T1 mapping was performed using a total of six variable-flip-angle three-dimensional spoiled gradient recalled echo sequences (flip angles: 2°, 5°, 10°, 15°, 20°, and 25°; repetition time/echo time, 5.5/2.1 ms; number of effective excitations, 0.7; spatial resolution, 2 mm × 2 mm × 4 mm; scan time, 3 minutes). The DCE-MRI acquisition consisted of a three-dimensional fast spoiled gradient recalled echo sequence to gain sufficient SNR, contrast, and temporal resolution. The following scan parameters were used: flip angle, 15°; repetition time/echo time, 3.6/1.0 ms; number of effective excitations, 0.7; spatial resolution, 2 mm × 2 mm × 4 mm; temporal resolution, 5.5 s; number of temporal frames, 56; pixel bandwidth, 326 Hz; acceleration factor, 2; and scan time, 6 minutes. Gadopentetate dimeglumine (Magnevist; Bayer HealthCare Pharmaceuticals) was administered intravenously to the patients at the end of the sixth frame (dose, 0.1 mmol/kg at a rate of 3 mL/second) followed by a 20-mL saline flush via a power injector (Spectris MR Injector; Medrad) at a rate of 3 mL/second.

Variable-flip-angle images, DCE-MRI images, and a bootstrapped population AIF measured in a region of interest in the carotid artery^11^ were distributed to each institution to use in their algorithm(s) to generate K^trans^ and v_e_ parameter maps for each patient.

Each patient had 6 regions of interest (ROIs)—contralateral and ipsilateral parotid glands, contralateral and ipsilateral submandibular glands, sublingual glands, and a primary gross tumor volume (GTV-P)—contoured on his or her pretreatment images by a radiation oncologist with 7 years of experience (A.S.R. Mohamed). Midtreatment and posttreatment images were deformably registered to the pretreatment images using a commercially available software program (Velocity AI, version 3.0.1; Varian Medical Systems). The deformation vector fields were exported from the deformation software and used with an in-house MATLAB code (MATLAB 2014b; MathWorks) to deform the ROIs and extract K^trans^ and v_e_ values from the six ROIs on each parameter map at the three time points. For each ROI, K^trans^ and v_e_ values were subjected to the same threshold constraints as in the DROs and then averaged.

### Statistical methods

A stratified permutation test was designed to determine whether the K^trans^ and v_e_ values from an algorithm for a specific DRO were generally ordered correctly in the DRO. Permutation tests work by rearranging data in many possible ways in order to estimate the sampling distribution for the test statistic. Algorithms were compared on a pairwise basis using a paired Wilcoxon rank-sum test to determine if the outputs of two algorithms were statistically different (R software package, version 3.3.1). Algorithms were split into two groups based on if spatial averaging was used on the DCE-MRI scans. The two groups were compared using a one-sided student’s t-test to determine if lower error on the DROs was calculated when spatial averaging was used. All p-values were adjusted using the Benjamini-Hochberg correction for multiple comparisons.

For patient DCE-MRI data, consistency of trends across algorithms was assessed using linear mixed effects models (R lme4 package, version 1.1.12) constructed for the differences between the pretreatment and midtreatment, pretreatment and posttreatment, and midtreatment and posttreatment quantitative metrics, and percent change in these three time differences. Two mixed effects models were created: one in which the algorithm was a fixed effect and the ROI was a random effect (Δ ~ algorithm + (1|ROI)) and one in which only the random effect of the ROI was included (Δ ~ 1 + (1|ROI)). A likelihood ratio test was performed for these two models to determine if the algorithm was a significant factor in the measured changes. All p-values were adjusted using the Benjamini-Hochberg correction for multiple comparisons (R, version 3.3.1). We used linear mixed effects models with likelihood ratio tests instead of ANOVA tests because in most comparisons we observed statistically different variances as determined using the Levene test, which violates one of the assumptions of ANOVA tests. Intraclass correlation coefficient is more appropriate for complete data sets^46^, so it was not applicable for this data set.

For all ROIs, patients were categorized as above or below the median values from a given algorithm using three different metrics: (1) each time point, (2) difference between time points, and (3) percent difference between time points. Krippendorff’s alpha was used to assess inter-algorithm reliability (R, irr package, version 0.84). We used Krippendorff’s alpha to compare algorithms because of its ability to handle missing data, which occurred because for some algorithms, all K^trans^ and v_e_ values were outside the threshold for a given patient’s ROI.

Trends within each algorithm were assessed using Spearman’s rank correlation coefficient (R, version 3.3.1). Spearman correlations were conducted using three different sets of time points: (1) all three time points, (2) only the pretreatment and midtreatment time points, and (3) only the pretreatment and posttreatment time points were evaluated. All p-values were adjusted using the Benjamini-Hochberg correction for multiple comparisons. For all statistical tests, p-values below 0.05 after adjustment were considered significant.

## Electronic supplementary material

Supplemental Data

## References

[1] Jemal A (2011). Global cancer statistics. CA: a cancer journal for clinicians.

[2] Howlader, N. *et al*. SEER Cancer Statistics Review, 1975–2014, National Cancer Institute. Bethesda, MD, https://seer.cancer.gov/csr/1975–2014/, based on November 2016 SEER data submission, posted to the SEER web site, April 2017.

[3] Bernstein JM, Bernstein CR, West CM, Homer JJ (2013). Molecular and cellular processes underlying the hallmarks of head and neck cancer. European archives of oto-rhino-laryngology.

[4] Horsman MR, Mortensen LS, Petersen JB, Busk M, Overgaard J (2012). Imaging hypoxia to improve radiotherapy outcome. Nature reviews clinical oncology.

[5] Houweling AC (2011). MRI to quantify early radiation-induced changes in the salivary glands. Radiotherapy and oncology.

[6] Juan CJ (2009). Perfusion characteristics of late radiation injury of parotid glands: quantitative evaluation with dynamic contrast-enhanced MRI. European radiology.

[7] Bernstein JM, Homer JJ, West CM (2014). Dynamic contrast-enhanced magnetic resonance imaging biomarkers in head and neck cancer: potential to guide treatment? A systematic review. Oral oncology.

[8] Noij DP (2015). Contrast-enhanced perfusion magnetic resonance imaging for head and neck squamous cell carcinoma: a systematic review. Oral oncology.

[9] Cheng CC (2013). Parotid perfusion in nasopharyngeal carcinoma patients in early-to-intermediate stage after low-dose intensity-modulated radiotherapy: evaluated by fat-saturated dynamic contrast-enhanced magnetic resonance imaging. Magnetic resonance imaging.

[10] Lee FK, King AD, Kam MK, Ma BB, Yeung DK (2011). Radiation injury of the parotid glands during treatment for head and neck cancer: assessment using dynamic contrast-enhanced MR imaging. Radiation research.

[11] Cooperative JHaNR-MD (2016). Dynamic contrast-enhanced MRI detects acute radiotherapy-induced alterations in mandibular microvasculature: prospective assessment of imaging biomarkers of normal tissue injury. Scientific reports.

[12] Huang W (2016). The Impact of Arterial Input Function Determination Variations on Prostate Dynamic Contrast-Enhanced Magnetic Resonance Imaging Pharmacokinetic Modeling: A Multicenter Data Analysis Challenge. Tomography.

[13] Yankeelov TE, Gore JC (2009). Dynamic Contrast Enhanced Magnetic Resonance Imaging in Oncology: Theory, Data Acquisition, Analysis, and Examples. Current medical imaging reviews.

[14] Yankeelov TE, Rooney WD, Li X, Springer CS (2003). Variation of the relaxographic “shutter-speed” for transcytolemmal water exchange affects the CR bolus-tracking curve shape. Magnetic resonance in medicine.

[15] Leach M (2012). Imaging vascular function for early stage clinical trials using dynamic contrast-enhanced magnetic resonance imaging. European radiology.

[16] Schabel MC, Parker DL (2008). Uncertainty and bias in contrast concentration measurements using spoiled gradient echo pulse sequences. Physics in medicine and biology.

[17] Yang C (2009). Reproducibility assessment of a multiple reference tissue method for quantitative dynamic contrast enhanced-MRI analysis. Magnetic resonance in medicine.

[18] Heisen M (2010). The influence of temporal resolution in determining pharmacokinetic parameters from DCE-MRI data. Magnetic resonance in medicine.

[19] Di Giovanni P (2010). The accuracy of pharmacokinetic parameter measurement in DCE-MRI of the breast at 3 T. Physics in medicine and biology.

[20] Sourbron SP, Buckley DL (2011). On the scope and interpretation of the Tofts models for DCE-MRI. Magnetic resonance in medicine.

[21] Sourbron SP, Buckley DL (2012). Tracer kinetic modelling in MRI: estimating perfusion and capillary permeability. Physics in medicine and biology.

[22] Othman AE (2016). Comparison of different population-averaged arterial-input-functions in dynamic contrast-enhanced MRI of the prostate: Effects on pharmacokinetic parameters and their diagnostic performance. Magnetic resonance imaging.

[23] Tofts PS, Kermode AG (1991). Measurement of the blood-brain barrier permeability and leakage space using dynamic MR imaging. 1. Fundamental concepts. Magnetic resonance in medicine.

[24] Heye T (2013). Reproducibility of dynamic contrast-enhanced MR imaging. Part I. Perfusion characteristics in the female pelvis by using multiple computer-aided diagnosis perfusion analysis solutions. Radiology.

[25] Huang W (2014). Variations of dynamic contrast-enhanced magnetic resonance imaging in evaluation of breast cancer therapy response: a multicenter data analysis challenge. Translational oncology.

[26] Beuzit L (2016). Dynamic contrast-enhanced MRI: Study of inter-software accuracy and reproducibility using simulated and clinical data. Journal of magnetic resonance imaging.

[27] Cron, G. O. *et al*. Bias and precision of three different DCE-MRI analysis software packages: a comparison using simulated data. *International Society for Magnetic Resonance in Medicine*. (Proc 22nd Annual Meeting ISMRM, Milan (abstract 4592)) (2014).

[28] Tofts PS (1997). Modeling tracer kinetics in dynamic Gd-DTPA MR imaging. Journal of magnetic resonance imaging.

[29] Tofts PS (1999). Estimating kinetic parameters from dynamic contrast-enhanced T(1)-weighted MRI of a diffusable tracer: standardized quantities and symbols. Journal of magnetic resonance imaging.

[30] *Quantitative Imaging Biomarkers Alliance*, https://www.rsna.org/QIBA/.

[31] Carletta J (1996). Assessing agreement on classification tasks: the kappa statistic. Computational linguistics.

[32] Krippendorff K (2004). Reliability in content analysis. Human communication research.

[33] Neuendorf, K. A. *The content analysis guidebook*. (Sage, 2002).

[34] Kim S (2010). Prediction of response to chemoradiation therapy in squamous cell carcinomas of the head and neck using dynamic contrast-enhanced MR imaging. American journal of neuroradiology.

[35] Van Cann EM (2008). Quantitative dynamic contrast-enhanced MRI for the assessment of mandibular invasion by squamous cell carcinoma. Oral oncology.

[36] Lee FK, King AD, Ma BB, Yeung DK (2012). Dynamic contrast enhancement magnetic resonance imaging (DCE-MRI) for differential diagnosis in head and neck cancers. European journal of radiology.

[37] Bisdas S (2010). An exploratory pilot study into the association between microcirculatory parameters derived by MRI-based pharmacokinetic analysis and glucose utilization estimated by PET-CT imaging in head and neck cancer. European radiology.

[38] Tofts PS, Berkowitz B, Schnall MD (1995). Quantitative analysis of dynamic Gd-DTPA enhancement in breast tumors using a permeability model. Magnetic resonance in medicine.

[39] Ashton E (2010). Quantitative MR in multi-center clinical trials. Journal of magnetic resonance imaging.

[40] Schabel MC, Morrell GR (2009). Uncertainty in T(1) mapping using the variable flip angle method with two flip angles. Physics in medicine and biology.

[41] Eklund A, Nichols TE, Knutsson H (2016). Cluster failure: Why fMRI inferences for spatial extent have inflated false-positive rates. Proceedings of the National Academy of Sciences of the United States of America.

[42] Galbraith SM (2002). Reproducibility of dynamic contrast-enhanced MRI in human muscle and tumours: comparison of quantitative and semi-quantitative analysis. NMR in biomedicine.

[43] Butterworth E, Jardine BE, Raymond GM, Neal ML, Bassingthwaighte JB (2013). JSim, an open-source modeling system for data analysis. F1000Research.

[44] Barboriak, D. P. QIBA_v6_Tofts_RevB, https://sites.duke.edu/dblab/qibacontent/.

[45] Barboriak, D. P. QIBA_v9_Tofts, https://sites.duke.edu/dblab/qibacontent/.

[46] Gelman, A. & Hill, J. *Data analysis using regression and multilevel/hierarchical models*. 45–46 (Cambridge University Press, 2006).

[47] Korporaal JG (2011). Phase-based arterial input function measurements in the femoral arteries for quantification of dynamic contrast-enhanced (DCE) MRI and comparison with DCE-CT. Magnetic resonance in medicine.

[48] *NordicNeuroLab*, http://www.nordicneurolab.com/.

[49] Li X (2008). Dynamic NMR effects in breast cancer dynamic-contrast-enhanced MRI. Proceedings of the National Academy of Sciences of the United States of America.

[50] Tudorica A (2016). Early Prediction and Evaluation of Breast Cancer Response to Neoadjuvant Chemotherapy Using Quantitative DCE-MRI. Translational oncology.

[51] Coolens C (2015). Automated voxel-based analysis of volumetric dynamic contrast-enhanced CT data improves measurement of serial changes in tumor vascular biomarkers. International journal of radiation oncology, biology, physics.

[52] Coolens C, Driscoll B, Moseley J, Brock KK, Dawson LA (2016). Feasibility of 4D perfusion CT imaging for the assessment of liver treatment response following SBRT and sorafenib. Advances in Radiation Oncology.

[53] Hormuth DA, Skinner JT, Does MD, Yankeelov TE (2014). A comparison of individual and population-derived vascular input functions for quantitative DCE-MRI in rats. Magnetic resonance imaging.

[54] Barnes SL, Whisenant JG, Loveless ME, Yankeelov TE (2012). Practical dynamic contrast enhanced MRI in small animal models of cancer: data acquisition, data analysis, and interpretation. Pharmaceutics.

