# Publisher Correction: A Multi-Institutional Comparison of Dynamic Contrast-Enhanced Magnetic Resonance Imaging Parameter Calculations

**DOI:** 10.1038/s41598-018-20639-z

**Published:** 2018-03-08

**Authors:** Joint Head and Neck Radiotherapy-MRI Development Cooperative, Rachel B. Ger, Abdallah S. R. Mohamed, Musaddiq J. Awan, Yao Ding, Kimberly Li, Xenia J. Fave, Andrew L. Beers, Brandon Driscoll, Hesham Elhalawani, David A. Hormuth, Petra J. van Houdt, Renjie He, Shouhao Zhou, Kelsey B. Mathieu, Heng Li, Catherine Coolens, Caroline Chung, James A. Bankson, Wei Huang, Jihong Wang, Vlad C. Sandulache, Stephen Y. Lai, Rebecca M. Howell, R. Jason Stafford, Thomas E. Yankeelov, Uulke A. van der Heide, Steven J. Frank, Daniel P. Barboriak, John D. Hazle, Laurence E. Court, Jayashree Kalpathy-Cramer, Clifton D. Fuller

**Affiliations:** 1 Department of Radiation Physics, The University of Texas MD Anderson Cancer Center, Houston, Texas USA; 2 The University of Texas MD Anderson Cancer Center UTHealth Graduate School of Biomedical Sciences, Houston, Texas USA; 3 Department of Radiation Oncology, The University of Texas MD Anderson Cancer Center, Houston, Texas USA; 4 Department of Clinical Oncology and Nuclear Medicine, Faculty of Medicine, University of Alexandria, Alexandria, Egypt; 5 Case Western Reserve University, Cleveland, OH USA; 6 University Hospitals, Cleveland, OH USA; 7 Department of Imaging Physics, The University of Texas MD Anderson Cancer Center, Houston, Texas USA; 8 Advanced Imaging Research Center, Knight Cancer Institute, Oregon Health & Science University, Portland, Oregon USA; 9 The International School of Beaverton, Beaverton, Oregon USA; 10 Athinoula A. Martinos Center for Biomedical Imaging, Massachusetts General Hospital/Division of Health Sciences & Technology, Massachusetts Institute of Technology, Charlestown, Massachusetts USA; 11 Techna Institute, University Health Network, Toronto, Ontario Canada; 12 Institute for Computational Engineering and Sciences, The University of Texas, Austin, Texas USA; 13 Department of Radiation Oncology, The Netherlands Cancer Institute, Amsterdam, The Netherlands; 14 United Imaging Healthcare America, Houston, Texas USA; 15 Department of Biostatistics, The University of Texas MD Anderson Cancer Center, Houston, Texas USA; 16 Radiation Medicine Program, Princess Margaret Cancer Centre, University Health Network, Toronto, Ontario Canada; 17 Department of Radiation Oncology, University of Toronto, Toronto, Ontario Canada; 18 Department of Otolaryngology Head and Neck Surgery, Baylor College of Medicine, Houston, Texas USA; 19 Department of Head and Neck Surgery, The University of Texas MD Anderson Cancer Center, Houston, Texas USA; 20 Department of Molecular and Cellular Oncology, The University of Texas MD Anderson Cancer Center, Houston, Texas USA; 21 Department of Radiology, Duke University Medical Center, Durham, North Carolina USA

Correction to: *Scientific Reports* 10.1038/s41598-017-11554-w, published online 11 September 2017

The original version of this Article incorrectly listed the authors individually rather than as part of the Joint Head and Neck Radiotherapy-MRI Development Cooperative.

This error has now been corrected in the PDF and HTML version of the Article.

