# Supplementary material for: A Multi-Institutional Comparison of Dynamic Contrast-Enhanced Magnetic Resonance Imaging Parameter Calculations
Source: Sci Rep. 2017 Sep 11;7:11185. doi: 10.1038/s41598-017-11554-w (PMC5593829; doi:10.1038/s41598-017-11554-w)
Supplement: Supplementary file 1 — Supplemental Data [file 41598_2017_11554_MOESM1_ESM.pdf]

# A Multi-Institutional Comparison of Dynamic Contrast-Enhanced Magnetic Resonance Imaging Parameter Calculations

Joint Head and Neck Radiotherapy-MRI Development Cooperative

Rachel B. Ger, Abdallah S. R. Mohamed, Musaddiq J. Awan**,** Yao Ding, Kimberly Li, Xenia J. Fave, Andrew L. Beers, Brandon Driscoll, Hesham Elhalawani, David A. Hormuth, II, Petra J. van Houdt, Renjie He, Shouhao Zhou, Kelsey B. Mathieu, Heng Li, Catherine Coolens, Caroline Chung, James A. Bankson, Wei Huang, Jihong Wang, Vlad C. Sandulache, Stephen Y. Lai, Rebecca M. Howell, R. Jason Stafford, Thomas E. Yankeelov, Uulke A. van der Heide, Steven J. Frank, Daniel P. Barboriak, John D. Hazle, Laurence E. Court, Jayashree Kalpathy-Cramer and Clifton D. Fuller

Supplemental Data

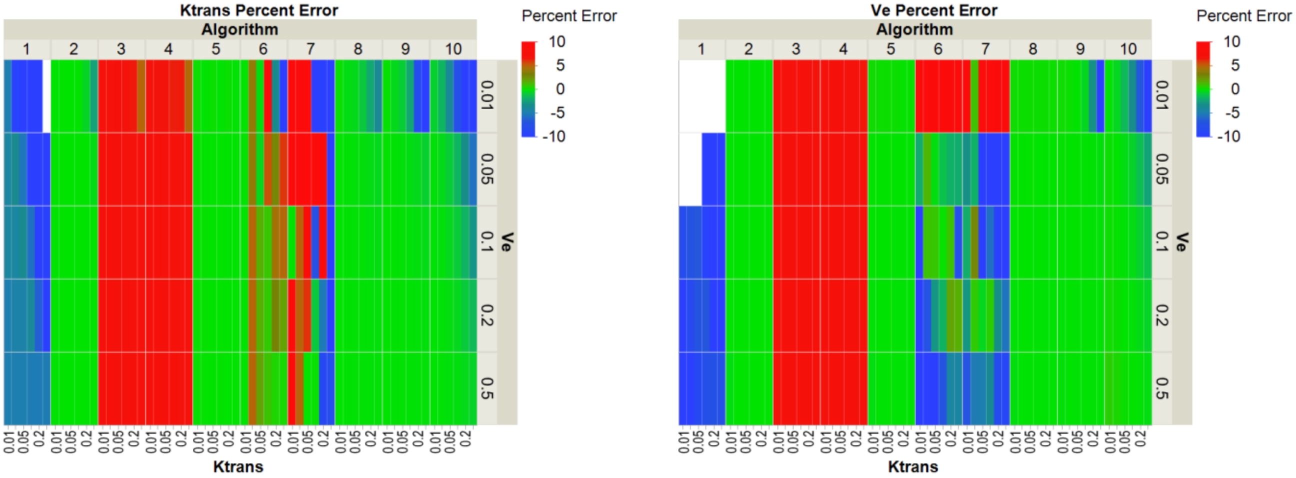


**Supplemental Figure 1.**

Heat maps of the percentage error for $K^{trans}$ (left) and $v_e$ (right) in the DRO without noise.

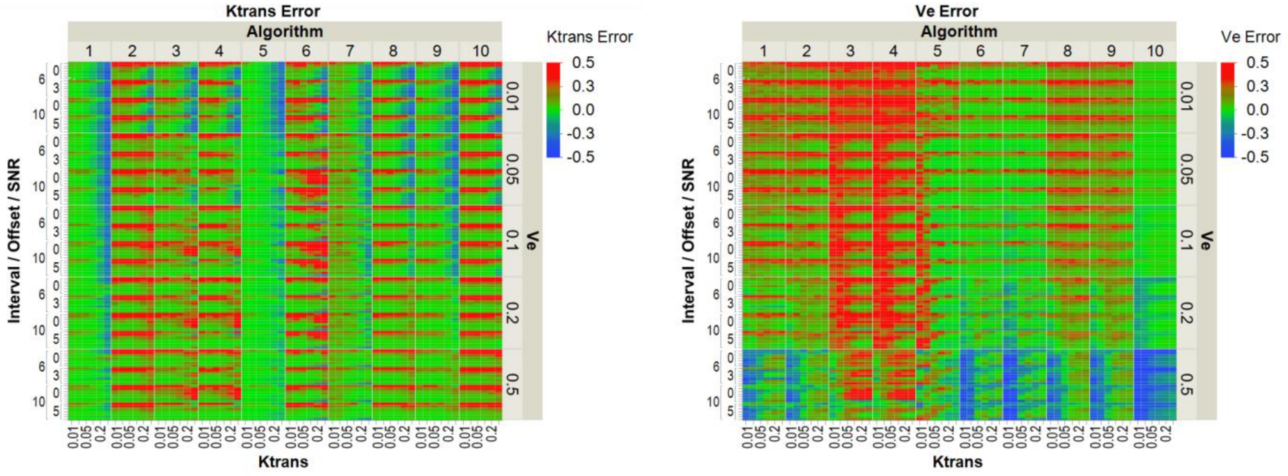


**Supplemental Figure 2.**

Heat maps of the error for $K^{trans}$ (left) and $v_e$ (right) in the 28 DROs with noise. Maximum error is defined as 0.5, and minimum error is defined as -0.5. Any differences between the measured and simulated values greater than 0.5 are mapped to 0.5 and any differences less than -0.5 are mapped to -0.5. (See the inset in Fig. 2 for all $K^{trans}$ and SNR values.)

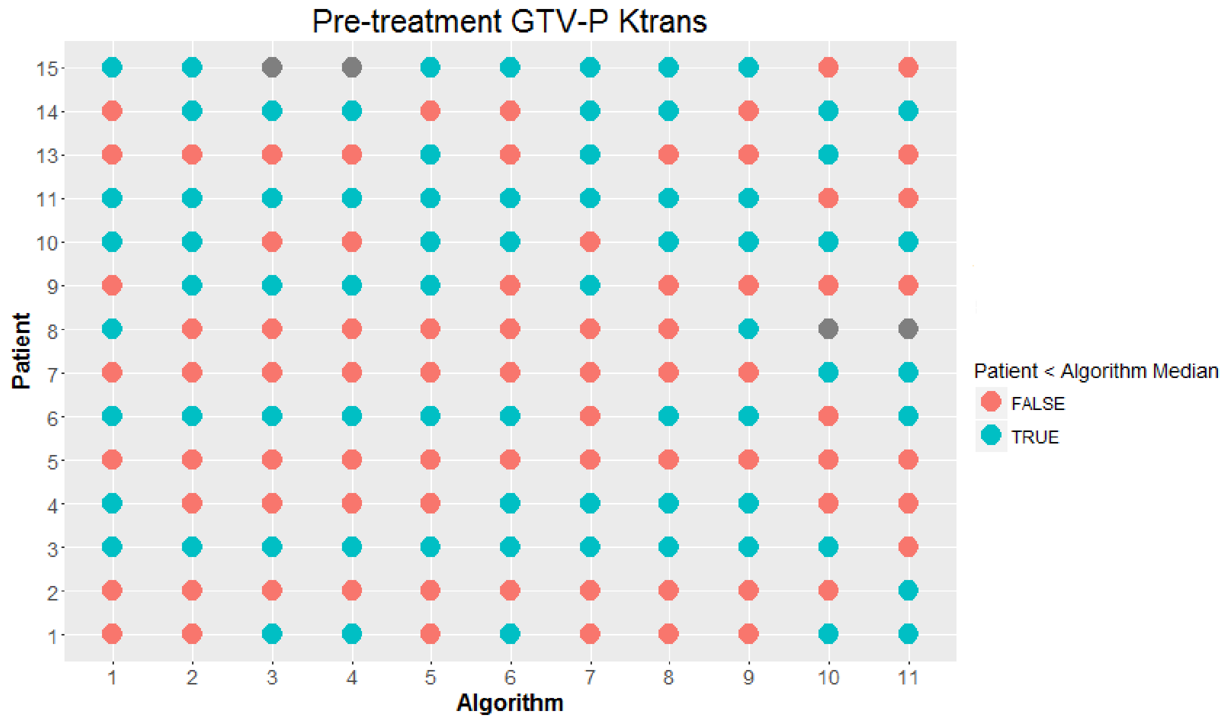


**Supplemental Figure 3.**

Demonstration of the Krippendorff's alpha test for pretreatment GTV-P. Red indicates that a patient's pretreatment GTV-P $K^{trans}$ value is above the median pretreatment GTV-P $K^{trans}$ value for that algorithm. Blue indicates that a patient's pretreatment GTV-P $K^{trans}$ value is below the median pretreatment GTV-P $K^{trans}$ value for that algorithm. Gray indicates that the patient's pretreatment GTV-P $K^{trans}$ value was outside the bounds of the threshold. Overall, algorithms do not agree in classifying if a patient's pretreatment GTVP $K^{trans}$ value is above or below the median for all patients.

**Description of MDA Model**

Pre-contrast T1 maps were generated from the variable flip angle patient data. T1 values were calculated for each voxel in MATLAB (R2013a; MathWorks, Natick, MA) by performing a nonlinear curve fit (using "lsqcurvefit") between signal intensity and the steady state signal equation for fast spoiled gradient echo sequences. Voxels with nonphysiological T1 values for soft tissue (T1<0.3s or T1>3.3s) were flagged and excluded from further analysis. Dynamic gadolinium concentration was calculated for the population AIF and each voxel of dynamic data which exceeded a minimum signal intensity threshold, assuming a relaxivity of 3.3/mM/s for Gd-DTPA and a baseline T1 value of 1600ms for blood at 3T. A hematocrit value of 41% was assumed in these analyses.

All dynamic Gd concentration curves were trimmed to align the beginning of enhancement in AIF and tissue concentration curves and to ensure a consistent number of dynamic frames. Voxels that yielded negative or complex Gd concentration were flagged and excluded from further analysis. DCE-MRI vascular parameters were calculated by fitting dynamic data to the Tofts and extended Tofts models.

**Description of MGH Model**

The method for the DRO images and patient images were similar. The mri_ms_fitparms command from Freesurfer (https://surfer.nmr.mgh.harvard.edu/fswiki/mri_ms_fitparms) was used to create T1 maps from the provided variable flip angle files. These T1 maps then had a 2D Gaussian blur applied on each axial slice, or on just the one slice in the case of the DROs. For the patients, a population AIF was used from the AIF spreadsheet provided. For the DROs, AIFs were averaged from the provided AIF ROIs. All images except for the noiseless DRO were subject to a 2D Gaussian filter applied to axial slices. Signal intensity was converted into Gd concentration using the provided parameters for repetition time, flip angle and relaxivity. Hematocrit was assumed at 45%. T1 parameters were determined voxel-by-voxel from the T1 map. $K^{trans}$ and $v_e$ values were fit using the two-parameter Tofts model. Fitting was achieved via the Nelder-Mead Simplex algorithm in Matlab, with a cost-function determined by subtracting the AUC between expected signal intensity and observed signal intensity. AUCs were determined by piecewise linear integration across the entire timespan of the scan. Voxels that fit to unreasonable $K^{trans}$ or $v_e$ values were set to -.01.
